# Outcomes of Implant Exchange and Latissimus Dorsi Flap Replacement After Breast Implant Complications

**DOI:** 10.1007/s00266-024-04107-w

**Published:** 2024-05-13

**Authors:** Mohamed F. Asal, Khaled E. Barakat, Ahmed Adham R. Elsayed, Ahmed T. Awad, Marc D. Basson

**Affiliations:** 1 Surgical Oncology and Breast Reconstruction Unit, Alexandria Faculty of Medicine, Alexandria, 21521 Egypt; 2 Department of Surgery, Northeast Ohio Medical University, 4209 State Route 44, Rootstown, OH 44272 USA; 3 Department of Anatomy and Neurobiology, Northeast Ohio Medical University, Rootstown, OH 44272 USA; 4 University Hospitals NEOMED Faculty Scholar, Cleveland, OH USA

**Keywords:** Implant exchange, Implant failure, Latissimus dorsi flap, Breast implant, Operation time

## Abstract

**Background:**

Immediate action is required to address some complications of implant-based reconstruction after mastectomy to prevent reconstruction failure. Implant exchange may be simple but poses the risk of further complications while autologous flap reconstruction seems more complex but may pose less subsequent risk. Which of these is preferable remains unclear.

**Methods:**

We reviewed thirty-two female breast cancer patients who had serious complications with their breast implants after post-mastectomy reconstruction. Latissimus dorsi flap (LDF) patients underwent explantation and immediate reconstruction with an LDF, while implant exchange (IE) patients underwent immediate implant removal and exchange with an expander followed by delayed reconstruction with silicon or immediately with a smaller size silicone implant.

**Results:**

LDF patients underwent a single operation with an average duration of care of 31 days compared to an average 1.8 procedures (*p*= 0.005) with an average duration of care of 129.9 days (*p *< 0.001) among IE patients. Seven IE (50%) had serious complications that required subsequent revision while no LDF patients required additional procedures. Patient overall satisfaction and esthetics results were also superior in the LDF group at six months.

**Conclusion:**

In patients who want to reconstructively rescue and salvage their severely infected or exposed breast implant, the LDF offers an entirely autologous solution. LDF reconstruction in this setting allows patients to avoid an extended duration of care, reduces their risk of complications, and preserves the reconstructive process.

**Level of Evidence III:**

The journal asks authors to assign a level of evidence to each article. For a complete description of Evidence-Based Medicine ratings, see the Table of Contents or the online Instructions for Authors at www.springer.com/00266.

## Introduction

Breast cancer is the most frequently diagnosed cancer in the world [1]. In the USA, it is the second most common cancer in women after skin cancer [2, 3] and the second-leading cause of cancer-related death among US women, behind lung cancer [3, 4].

The most common method of reconstruction after mastectomy is implant-based reconstruction [5–7]. Indeed, reconstruction via manufactured implants typically yields good results and high patient satisfaction. However, such procedures may sometimes result in infection or implant exposure. The more common use of postoperative radiotherapy has raised the rates of implant failure and capsular contracture [8] while wound infections are more frequent in such patients [9].

A patient with a wound infection after implant reconstruction may be treated without surgery [10], but surgical options should be considered if the infection does not subside. A surgical emergency might arise from implant exposure due to the risk of implant loss [6, 11, 12]. Exchange of implants is typically necessary for patients who have exposed implants, but these procedures may pose substantial challenges, particularly if they occur in patients who also have adverse effects of radiation treatment [13].

Complete mastectomy obviously prevents the breast reconstruction that the patient had hoped for, while replacing the infected implant with an expander followed by delayed reconstruction reduces patient satisfaction because of the delay [14]. Immediate implant exchange may require a smaller size silicone implant, which may also reduce satisfaction or involve subsequent procedures to match the other breast. Implant explantation and reconstruction by an autologous flap is a more complex option.

Reconstruction with an autologous flap has many options such as deep inferior epigastric artery perforator (DIEP) flap, traverse rectus abdominal muscle (TRAM) flap, and latissimus dorsi (LD) flap. DIEP free flap may be considered for reconstruction but it is not the preferred method in such a difficult situation as it offers free flap complications to these complicated patients [15]. Also, the TRAM flap may cause infection in the abdomen. LD flap was the preferred method to do the reconstructions. The latissimus dorsi flap is one of the most versatile and reliable means for breast reconstruction [16]. However, it is typically the last resort for reconstruction because the patient will lose the ability to achieve any further reconstruction using this method.

In the case of a previously failed implant, we speculated that the risk of losing further reconstruction and the more invasive nature of the procedure would be offset by the chance to definitively manage the complication with an LD flap. Thus, we hypothesized that explantation with immediate conversion to a wholly pedicled autologous reconstruction would salvage potentially failed breast reconstructions without risking recurring further complications. We retrospectively compared patients who underwent explantation and immediate reconstruction with an LD flap replacement (denoted “LDF” for latissimus dorsi flap) to patients who underwent either an immediate implant exchange with a smaller size of silicon or an exchange with an expander (denoted “IE” for implant exchange), followed by delayed reconstruction with silicon based on the duration of care, number of surgical procedures, operative time, hospital stay, recovery period, and the incidence of complications requiring further revisional procedures.

## Materials and Methods

### Patients

In this retrospective study, female breast cancer patients presenting with a serious implant-related complication following post-mastectomy reconstruction while hospitalized in Surgical Oncology Unit, Alexandria Main University Hospital, between August 2021 and February 2023 were included.

LDF patients underwent explantation and immediate reconstruction with an LD flap replacement. IE patients underwent either an immediate implant exchange with a smaller size of silicon or exchange with an expander, followed by delayed reconstruction with silicon. All options were offered to all patients after the failure of the first implant. The patients had the option to choose between implant replacement, LDF replacement, or implant removal with no further reconstruction, and the choice of procedure was determined solely by patient preference.

All patients in both groups underwent lavage with 4 bottles mixture of 500 ml saline, 2 ampules of gentamicin 40mg/1ml & 30 cm^3^ betadine. We have used transverse or oblique LD according to the available tissue. We removed the implant and replaced it with a latissimus dorsi flap as previously described [16].

We compared LDF patients to IE based on the duration of care, number of surgical procedures, operative time, hospital stay, and recovery period (defined as the duration between the surgical procedure and final resumption of normal daily activity which is determined by a routinely used follow-up questionnaire filled out by the patients), and incidence of complications requiring revision surgical procedure. A routine esthetic and satisfaction assessment is performed at our institution on all patients at the 6-month follow-up visit after their procedure. The assessment is based on the patient’s perspective using a survey instrument previously described by Tzafetta [17]. Breast shape and contour, contralateral match, patient satisfaction, effect on sexual life, effect on social life, and the overall result are graded by the patient as excellent, good, fair, or poor. We compared the results of this assessment in the LDF and IE groups after 6 months.

The LDF group had an average follow-up period of 8.2 months (range 6–10 months), while the IE group had an average follow-up period of 15.14 months (range 11–20 months). All follow-ups were via in person visits in the clinic.

### Statistical Analysis

Statistical analysis was conducted using SPSS v26 (IBM Inc., Chicago, Illinois, USA). The Shapiro–Wilks test was used to determine whether the data distribution was normal. Quantitative parametric data were presented using the mean and standard deviation (SD). The median and the interquartile range (IQR) were employed to display numerical nonparametric data. The qualitative features were illustrated using frequency and percentages (%). We used Chi-square tests to compare categorical variables, with Fisher’s exact or Monte Carlo correction if more than 20% of the cells had an expected count less than 5. We used a student t-test to compare normally distributed quantitative variables and a Mann–Whitney test to compare abnormally distributed quantitative variables.

## Results

**Fig. 1:**
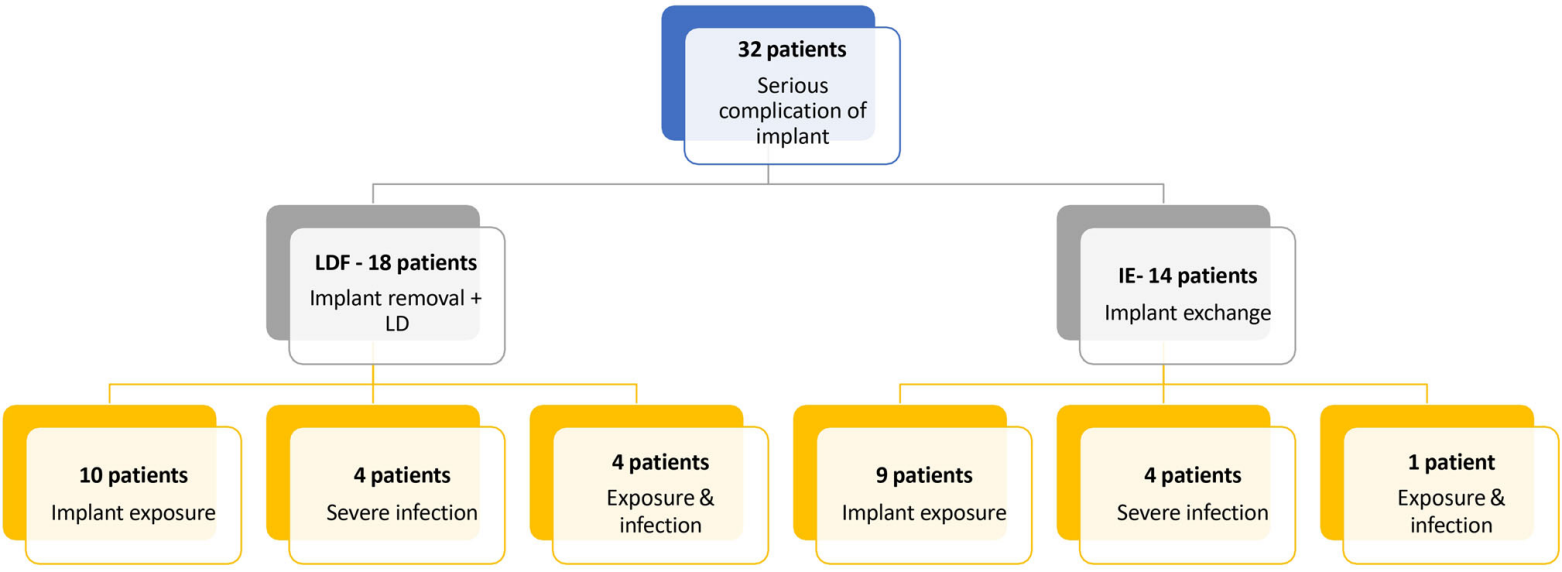
Classification of the patients in both groups according to the complications.

We identified 32 patients across both groups who had a preoperative serious implant-related complication following post-mastectomy reconstruction (Fig. 1). 47% had undergone preoperative radiotherapy as part of their treatment before their implant complication. This did not differ statistically between the two groups.

Among LDF patients (18 cases), the implant was exposed in ten patients without infection, and the patient requested to move to implant removal and LD flap replacement as their salvage procedure. Four patients had developed serious infections that could not be treated with antibiotics alone although the implant itself was not exposed. Reconstructive salvage was not considered to be prohibited by the presence of pus. Patients were kept on antibiotics after the surgical procedure depending on culture results until all clinical signs of infection disappeared. Four patients required immediate urgent surgical intervention due to simultaneous severe infection and implant exposure (Fig. 1).

Among IE patients (14 cases), the implant was only exposed in 9 patients. Four patients developed serious infections without implant exposure. One patient had a severe infection and implant exposure simultaneously. Nine of the 14 patients underwent implant exchange with a smaller size of silicon. The rest of the patients underwent implant removal and expander placement and then delayed reconstruction with silicon again after a mean of 3 months (Fig. 1).

Perioperative data showed no significant differences between the groups regarding age, diabetes mellitus (DM), hypertension (HTN), axillary status, or smoking or alcohol use. LDF patients did have a statistically significantly higher BMI than IE patients (*p*= 0.007*) (Table 1).

**Table 1:** Preoperative characteristics of the two groups

Comparison point	LDF (*n*=18)	IE (*n*=14)	Test of sig.	*P* value
*Age (years)*				
Min.–Max.	41.0–73.0	43.0–57.0	*t*= 1.158	0.258
Mean ± SD.	52.50 ± 8.95	49.79 ± 3.83		
Median (IQR)	51.0 (45.0–60.)	49.50 (47.0–52.0)		
*Past medical history*				
Positive	10 (55.5%)	8 (57.1%)	*χ*^2^=0.008	0.928
Negative	8 (44.4%)	6 (42.9%)		
*BMI (kg/m*^*2*^*)*				
Min.–Max.	31.40–54.90	28.80–38.90	*t*= 2.884^*^	0.007^*^
Mean ± SD.	37.47 ± 5.66	32.54 ± 3.37		
Median (IQR)	36.30 (33.70–40.20)	31.50 (29.80–34.20)		
*Axilla status*				
Negative	7 (38.9%)	5 (35.7%)	*χ*^2^= 0.034	0.854
Positive	11 (61.1%)	9 (64.3%)		
*Radiotherapy*				
Positive	8 (44%)	7 (50%)	*χ*^2^=0.098	0.755
Negative	10 (56%)	7 (50%)		
*Habits*				
No smoking	16 (88.9%)	13 (92.9%)	*χ*^2^=0.146	^FE^*p*=1.000
Smoking	2 (11.1%)	1 (7.1%)		
Alcohol use	0 (0.0%)	0 (0.0%)		

*IQR* Interquartile range, *SD* Standard deviation, *t* Student *t*-test, *U* Mann–Whitney test, *χ*2 Chi-square test

^*^Statistically significant at *p* ≤ 0.05

**Fig. 2:**
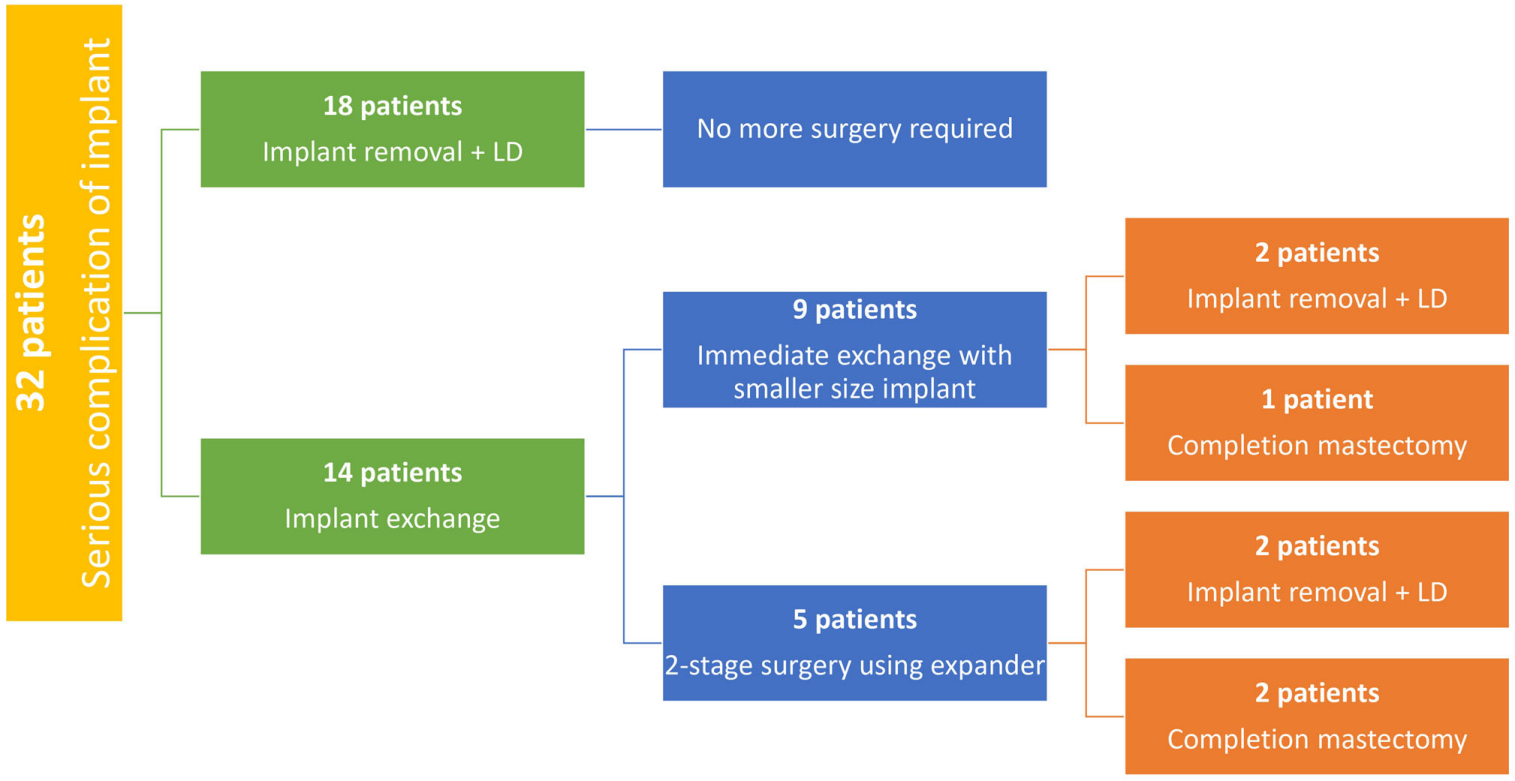
Classification of the patients according to procedures and results

Among LDF patients, eighteen underwent salvage of unilateral pre-pectoral silicon implantation with explantation of the silicon and immediate reconstruction with LD flap replacement. None of these patients required further revision surgery. Of the 14 IE patients, 9 underwent immediate exchange with a smaller size implant and 5 underwent two-stage surgical procedures using a tissue expander at the first stage. Seven (50%) out of the 14 patients had a serious complication (infection or exposure) that required further revision surgical procedure. Four patients underwent implant removal plus LDF. Three patients requested an implant removal without further reconstruction in frustration at their series of complications (Fig. 2).

After the first surgery, the two groups differed substantially in operative time and hospital stay. IE patients had shorter operative times and shorter hospital stays (Table 2).

**Table 2:** Comparison between the two groups after the first operation

Comparison point	LDF (*n*=18)	IE (*n*=14)	Test of sig.	*P* value
First operation time (min.)				
Min.–Max.	110.0–190.0	51.0–66.0	*U* = 0.00^*^	<0.001^*^
Mean ± SD.	143.61 ± 23.25	58.71 ± 4.56		
Median (IQR)	137.50(125.0–165.0)	58.50 (55.0–62.0)		
First operation hospital stay (days)				
Min.–Max.	2.0–2.0	1.0–1.0	*U* =0.00^*^	<0.001^*^
Mean ± SD.	2.0 ± 0.0	1.0 ± 0.0		
Median (IQR)	2.0	1.0		

*IQR* Interquartile range, *SD* Standard deviation, *U* Mann–Whitney test

^*^Statistically significant at *p* ≤ 0.05

However, these findings were very different when each patient’s complete course was considered. LDF patients underwent a single operation with an average duration of care of 31 days compared to an average 1.8 procedures (*p*= 0.005) with an average duration of care of 129.9 days (*p*<0.001) among IE patients (Table 3). Although the initial surgical procedure had been shorter for IE patients, total operative time did not differ significantly when the operative time for all relevant surgical procedures was summed. Similarly, while IE patients initially had shorter hospital stays (Table 2), the total hospital stay for LDF patients averaged 2±0 days in comparison a total hospital stay requirement of 3.6±1.6 days for IE patients (*p*=0.005). In addition, the average recovery period between the surgical procedure and ultimate return to normal daily activity was substantially shorter in LDF patients than IE patients (23.1 ± 4.06 days vs. 35.7 ± 15.97 days, *p*<0.05). Perhaps most importantly, seven IE patients (50%) required a revisional surgical procedure due to subsequent major complications, while no LDF patients experienced this (*p*<0.0001) (Table 3).

**Table 3:** Comparison between the two groups regarding entire operative journey data

Comparison point	LDF (*n*=18)	IE (*n*=14)	Test of sig.	*P* value
Duration of care** (Days)				
Min.–Max.	23.0–47.0	29.0–356.0	*U*= 34.0^*^	<0.001^*^
Mean ± SD.	31.0 ± 5.46	129.93 ± 115.65		
Median (IQR)	30.50 (27.0–33.0)	90.50 (37.0–217.0)		
*Number of surgical procedures*				
Min.–Max.	1.0–1.0	1.0–3.0	*U*= 54.0^*^	0.005^*^
Mean ± SD.	1.0 ± 0.0	1.79 ± 0.80		
Median (IQR)	0.0	2.0 (1.0–2.0)		
Operative time (Min.)				
Min.–Max.	110.0–190.0	45.0–272.0	*U*= 113.0	0.639
Mean ± SD.	143.61 ± 23.25	128.71 ± 78.37		
Median (IQR)	137.50(125.0–165.0)	123.50 (53.0–197.0)		
Hospital stay (Days)				
Min.–Max.	2.0–2.0	2.0–6.0	*U*= 54.0^*^	0.005^*^
Mean ± SD.	2.0 ± 0.0	3.57 ± 1.60		
Median (IQR)	2.0	4.0 (2.0–4.0)		
Recovery period*** (Days)				
Min.–Max.	17.0–34.0	16.0–59.0	*t*= 2.881^*^	0.012^*^
Mean ± SD.	23.11 ± 4.06	35.71 ± 15.97		
Median (IQR)	22.50 (20.0–25.0)	36.50 (20.0–53.0)		
Incidence of complications that needed a revision surgical procedure	0	7 cases (50%)	*Χ*^2^=11.520^*^	0.001^*^

*IQR* Interquartile range, *SD* Standard deviation, *t* Student *t*-test, *U* Mann–Whitney test, *χ*^2^ Chi-square test

^*^Statistically significant at *p* ≤ 0.05

^**^Duration of care represents the duration since the complication event happened until final discharge from the hospital after the last surgical procedure.

^***^Recovery period is the duration from the surgical procedure until ultimate return to normal daily activity

Comparison of esthetic assessment and satisfaction between patients from the LDF and IE groups revealed significant differences in sexual life, social life, and overall results in favor of the LDF group (Table 4).

**Table 4:** Esthetic and satisfaction assessment of the patients in both the LDF and IE groups after 6 months

Assessment item	I (Excellent)		II (Good)		III (Fair)		IV (Poor)			
	LDF group	IE group	LDF group	IE group	LDF group	IE group	LDF group	IE group	*X*^2^	*P* VALUE
Breast shape and contour	16 (88.9%)	8 (57.1%)	2 (11.1%)	5 (35.7%)	0 (0%)	0 (0%)	0 (0%)	1 (7.1%)	4.307	^MC^*p*=0.058
Contralateral match	4 (22.2%)	7 (50.0%)	4 (22.2%)	5 (35.7%)	8 (44.4%)	1 (7.1%)	2 (11.1%)	1 (7.1%)	6.347	^MC^*p*=0.083
Patient Satisfaction	14 (77.8%)	6 (42.9%)	4 (22.2%)	7 (50.0%)	0 (0%)	1 (7.1%)	0 (0%)	0 (0%)	4.415	^MC^*p*=0.086
Effect on sexual life	13 (72.2%)	4 (28.6%)	2 (11.1%)	9 (64.3%)	3 (16.7%)	1 (7.1%)	0 (0%)	0 (0%)	9.582^*^	^MC^*p*=0.006^*^
Effect on social life	16 (88.9%)	6 (42.9%)	1 (5.6%)	7 (50.0%)	1 (5.6%)	1 (7.1%)	0 (0%)	0 (0%)	8.688^*^	^MC^*p*=0.008^*^
Overall result	18 (100%)	8 (57.1%)	0 (0%)	5 (35.7%)	0 (0%)	0 (0%)	0 (0%)	1 (7.1%)	9.582^*^	^MC^*p*=0.003^*^

*χ*2 Chi-square test, *MC* Monte Carlo

^*^Statistically significant at *p* ≤ 0.05

## Discussion

Post-mastectomy implant reconstruction can lead to severe complications, requiring removal and extended recovery time. Attempting salvage without removing the implant might prolong and complicate the patient’s duration of care and increase the psychological burden. Thus, salvage commonly involves implant exchange either immediately or as a two-stage delayed reconstruction. However, implant exchange may itself require further procedures or lead to further complications. Therefore, we compared two management techniques: implant removal and LD flap reconstruction versus immediate or delayed implant exchange. Although LD flap reconstruction was initially a somewhat longer procedure, our retrospective analysis suggested that patients reconstructed with an LD flap after initial implant failure needed fewer surgical procedures, had fewer total days in the hospital, had substantially fewer complications that needed surgical intervention, and eventually returned to their normal daily activity more rapidly than patients undergoing implant exchange.

The average patients’ BMI in both LDF and IE groups was high, but the LDF patients’ BMI was significantly higher than that of IE patients. Although this did result in a significant difference between the two groups in this non-randomized retrospective study, one would if anything expect a higher complication rate in obese patients [18]. Thus, this difference cannot explain our results. Indeed, our results with LDF in such patients align with a previous description by Schwartz [19] of the use of the muscle-sparing latissimus dorsi flap in eleven morbidly obese patients seeking reconstructive salvage of infected implants. Schwartz reported that 8 of his 11 patients developed wound infections, and three had wound breakdown that required outpatient wound care. He had no complication related to the flap that needed further surgical intervention, but only one of his patient’s required a surgical revision, and that only was at the patient’s request for improved symmetry. In comparison, none of our LDF patients required revision, while our non-operative seroma rates were 7 out of 18 (38.9%) which required aspiration and wound dehiscence rates were 2 out 18 (11.1%). These contrasts quite favorably with the 50% revision rate in our series of implant exchange patients. Among the IE patients, four patients underwent second implant failure which resulted in implant removal plus LD flap. Also, three further patients from the IE group requested an implant removal without further reconstruction which was extremely difficult for these patients who had originally sought breast reconstruction.

Radiotherapy used as part of the breast cancer treatment may contribute to the relative avascularity of the tissue bed [9]. This is likely synergistic with postoperative chemotherapy with the radiation effect in its deleterious effects on immunity and wound healing [9]. Augmenting blood flow to the area with a well-perfused autologous tissue flap such as an LDF can aid in treating residual infection following implant removal. The use of thoracodorsal artery in the LDF provides a reliable vascular supply that uncommonly experiences ischemic complications even in high risk patients who are smokers or have diabetes [16].

Because IE patients had complications, they also required subsequent surgical procedures. This substantially increased the total hospital length of stay for these patients in comparison with the experience of the LDF patients. Prolonged LOS imposes a higher risk of additional infection [20, 21], which can result in further complications. Therefore, the duration from the surgical procedure until the patients restore their normal daily activity was substantially longer among IE patients than LDF patients. This is likely not only to increase cost [22] but also to impose substantial psychological stress on IE patients, who are already likely to exhibit impairments in self-esteem and body image after breast cancer diagnosis and mastectomy [23]. Furthermore, the increased burden on the healthcare system of additional procedures and hospital stays and prolonged recovery may overburden the healthcare system by increasing the cost and interfering with access to care for other patients who may need hospital resources like beds or operating rooms [24].

While many would remove the implant without continuing with reconstruction in cases of severe infection [11], others have suggested that tissue reconstruction may be safe in such cases [19, 25] while implant exchange has also been described [26]. Simply halting reconstruction may be safe but it leaves the patient without a breast at a psychologically critical time. This series suggests that implant exchange is indeed risky in such patients, but that tissue reconstruction may be a safe alternative for patients wishing to continue with reconstruction.

The study is limited by its retrospective nature. Patients were not randomized between the two procedures but rather chose based on their own expressed preference which procedure to undergo, so it is possible that this might in some fashions have biased our results. However, the two groups did seem overall comparable in risk factors except for the higher BMI among LDF patients that if anything should have worsened their results. Although we did ask the patients about their perspective following the procedures and a comparison was done between the LDF and IE groups after 6 months, the study is limited to the data that we collected at the six-month time point. Future work may explore longer-term patients’ perspectives further. All operations were done in the same medical center, although by different surgeons, so these results might not extrapolate as well to other hospital systems with different medical practices.

## Conclusion

In patients who want to reconstructively rescue and salvage their severely infected or exposed breast implant, the latissimus dorsi flap offers one of the safest procedures following complicated outcomes. This entirely autologous solution allows patients to avoid an extended duration of care, reduces their risk of complications, and preserves the reconstructive process. Indeed, this approach may be particularly suitable for the high body mass index patient with extra tissues along their sides and in the LD area for a definitive autologous reconstruction.
