# Correction: Outcomes of Implant Exchange and Latissimus Dorsi Flap Replacement After Breast Implant Complications

**DOI:** 10.1007/s00266-025-04721-2

**Published:** 2025-02-13

**Authors:** Mohamed F. Asal, Khaled E. Barakat, Ahmed Adham R. Elsayed, Ahmed T. Awad, Marc D. Basson

**Affiliations:** 1 Surgical Oncology and Breast Reconstruction Unit, Alexandria Faculty of Medicine, Alexandria, 21521 Egypt; 2 Department of Surgery, Northeast Ohio Medical University, 4209 State Route 44, Rootstown, OH 44272 USA; 3 Department of Anatomy and Neurobiology, Northeast Ohio Medical University, Rootstown, OH 44272 USA; 4 University Hospitals NEOMED Faculty Scholar, Cleveland, OH USA

**Correction to: Aesth Plast Surg (2024) 48:4945–4952** 10.1007/s00266-024-04107-w

The original online version of this article was revised to indicate that Mohamed F. Asal and Khaled E. Barakat should be considered co-first authors of the article.

The original article has been corrected.

